# Expression of T-Cell Receptor β-Chain mRNA and Protein in γ/δ T-Cells from Euthymic and Athymic Rats: Implications for T-Cell Lineage Divergence

**DOI:** 10.1155/2000/51534

**Published:** 2000

**Authors:** Astrid Bischof, Jung-Hyun Park, Thomas Hünig

**Affiliations:** 1 lnstitute for Virology and Immunobiology Versbacher Str. 7 Würzburg D-97078 Germany; 2 Peptide Engineering R.U. Korea Research Institute of Bioscience and Biotechnology Yusong Taejon Korea

**Keywords:** γ/δ T-cell, lineage decision, TCR, thymus

## Abstract

The relationship between α/β and γ/δ T-cell lineages was studied in rats using RT-PCR analysis of TCRβ transcripts in γ/δ T-cell hybridomas and an intracellular staining technique to detect TCRβ protein in primary γ/δ T-cells. We report the presence of functional TCRβ transcripts in 2/9 γ/δ T-cell hybridomas. About 15 % of peripheral γ/δ T-cells and thymocytes also express TCRβ protein, giving a minimum estimate for successful Tcrb rearrangement based on *ex* *vivo* single cell analysis. In athymic rats, γ/δ T-cells expressing intracellular β protein are present but at a lower frequency than in euthymic controls, suggesting that in the thymus, more γ/δ T-cell precursors pass through a stage where functional β rearrangement has occurred than in extrathymic sites. Analysis of TCR expression in purified transitory immature CD4^-^8^+^ (iCD8SP) thymocytes and their spontaneously developing CD4^+^8^+^ (DP) progeny showed that TCRγ mRNA is expressed in iCD8SP cells but not in their immediate DP progeny that reinitiate RAG-1 transcription and commence α/βTCR expression. We conclude that rat γ/δ T cells can separate from the α/β lineage after TCRβ expression, but not after entry into the DP compartment.


*Developmental Immunology*, 2000, Vol. 8(1), pp. 19-30
Reprints available directly from the publisher
Photocopying permitted by license only

© 2000 OPA (Overseas Publishers Association)
N.V. Published by license under the
Harwood Academic Publishers imprint,
part of The Gordon and Breach Publishing Group.
Printed in Malaysia

### Expression of T-Cell Receptor β-Chain mRNA and Protein in γ/δ T-Cells from Euthymic and Athymic Rats: Implications for T-Cell Lineage Divergence

ASTRID BISCHOF[a], JUNG-HYUN PARK[b] and THOMAS HÜNIG[a]*

[a]*Institute for Virology and Immunobiology, Versbacher Str. 7, D-97078 Würzburg, Germany and* [b]*Peptide Engineering R.U., Korea Research Institute of Bioscience and Biotechnology, Yusong, Taejon, Korea*

*(Received October 29, 1998; In final form March 17, 1999)*

The relationship between α/β and γ/δ T-cell lineages was studied in rats using RT-PCR analysis of TCRβ transcripts in γ/δ T-cell hybridomas and an intracellular staining technique to detect TCRβ protein in primary γ/δ T-cells. We report the presence of functional TCRβ transcripts in 2/9 γ/δ T-cell hybridomas. About 15 % of peripheral γ/δ T-cells and thymocytes also express TCRβ protein, giving a minimum estimate for successful Tcrb rearrangement based on *ex vivo* single cell analysis. In athymic rats, γ/δ T-cells expressing intracellular β protein are present,but at a lower frequency than in euthymic controls, suggesting that in the thymus, more γ/δ T-cell precursors pass through a stage where functional β rearrangement has occurred than in extrathymic sites. Analysis of TCR expression in purified transitory immature $CD4^-8^+$ (iCD8SP) thymocytes and their spontaneously developing $CD4^+8^+$ (DP) progeny showed that TCRγ mRNA is expressed in iCD8SP cells but not in their immediate DP progeny that reinitiate RAG-1 transcription and commence α/βTCR expression. We conclude that rat γ/δ T cells can separate from the α/β lineage after TCRβ expression, but not after entry into the DP compartment.

*Keywords:* γ/δ T-cell, lineage decision, TCR, thymus

*Abbreviations:*DP, CD4,8 double-positive, i.c., intracellular, (i)SP, (immature) single-positive, pTCR, pre-T-cell receptor, TN, triple-negative

### INTRODUCTION

In all vertebrate species examined, T-cells can be subdivided into "α/β" and "γ/δ" subclasses based on the expression of TCR heterodimers encoded by distinct rearranging loci. Although some overlap in function and specificity between the two subsets have been reported, major differences in TCR structure, repertoire diversity and anatomical location indicate distinct functions of both subsets within the immune system. In humans and rodents, γ/δ T-cells appear first in ontogeny but are rapidly overtaken in number by the major population of α/β T-cells (Havran and Allison, 1988; Itohara et al., 1989; Lawetzky et al., 1990;

* Corresponding author: Tel. 0049–931–201–3951, Fax: 0049–931–201–2243, e-mail: huenig@vim.uni-wuerzburg.de

Kühnlein et al., 1995). Both α/β and γ/δ T-cells are mainly produced in the thymus, although extrathymic maturation of both subsets is also observed in athymic mice and rats (Hünig, 1983; Matis et al., 1987; Hünig et al., 1989; Lake et al., 1991).

Most of the currently available data on the lineage relationship of α/β and γ/δ T-cells are derived from the mouse model, where genetic and serologic tools are most advanced (for recent reviews, see Fehling and von Boehmer 1997; Kang and Raulet 1997; Robey and Fowlkes 1998). There is general consensus in this system that precursors for both T-cell subsets are present within the early "triple negative" (TN) thymocyte population that lacks surface expression of CD4, CD8 and TCR molecules (Fowlkes et al., 1985; Scollay et al., 1988), and that lineage separation occurs before progression to the CD4,8 "double positive" (DP) compartment, where γ chain transcription is terminated (Wilson et al., 1994, 1996) and most delta loci are deleted as a result of α rearrangements (Chien et al., 1987; Malissen et al., 1992). Although the potential of phenotypically defined "late" subsets of mouse TN thymocytes to generate both T-cell subsets *in vitro* (Petrie et al., 1992, Godfrey et al., 1993) and *in vivo* (Petrie et al., 1992) does not exclude earlier lineage separation, two lines of evidence argue convincingly that differentiation events initiating lineage-specific TCR gene expression of α/β and γ/δ T-cells can occur within the same cell: Mature α/β T-cells not only contain γ and δ rearrangements (Saito et al., 1984, Garman et al., 1986, Livac et al., 1995, Nakajima et al., 1995), but these are depleted of in-frame joins, presumably as a result of γ/δ divergence (Dudley et al., 1995; Kang et al., 1995; Livac, et al., 1995); and conversely, β rearrangements were observed in γ/δ T-cells and thymocytes, although the reported selection for in-frame joins (Dudley et al., 1994, 1995; Burtrum et al., 1996) is controversial (Vicari et al., 1996). This latter issue is of particular interest because an overrepresentation of in-frame β rearrangements would suggest that even after "β selection", that is the rapid numerical expansion initiated in late TN thymocytes containing productive TCR β rearrangements (Mombaerts et al., 1992; Shinkai et al., 1992; Mallick et al., 1993) and an invariant pTCRα chain (Fehling et al., 1995) before entry into the DP subset, the dual potential for lineage decision is maintained.

Nothing is known about the relationship between α/β and γ/δ T-cell lineages in rats. Earlier work from our laboratory has indicated similarities between mice and rats in the ontogenetic appearance of the two subsets in the periphery (Lawetzky et al., 1990), and in the generation of dendritic epidermal T-cells bearing a highly conserved canonical γ/δ TCR (Kühnlein et al., 1996). Differences exist, however, regarding γ/δ T-cell representation in the gut, and the predominance of CD8α/β expression on peripheral rat, but not mouse γ/δ T-cells (Kühnlein et al., 1994). Studies on lineage relationship during thymic development are hampered in the rat by the absence of CD25/CD44-defined subsets during the TN stage and by insufficient sequence information on the TCR loci undergoing rearrangements. In the present study, we therefore analyzed mRNA expression at the level of γ/δ T-cell hybridomas to investigate the presence of alternate lineage transcripts, and visualized intracellular TCRβ protein at the level of individual γ/δ T-cells and thymocytes with the help of a TCRβ-specific mAb suitable for intracellular staining. The results indicate that in rats, γ/δ T-cells can separate from the common pathway up to the stage defined by TCRβ expression, but not after transition into the DP compartment.

### RESULTS

### Expression of TCRβ mRNA in rat γ/δ T-cells

In the rat, α/β and γ/δ T-cells are identified by the mAb R73 directed to a constant determinant of the TCRβ chain (Hünig et al., 1989), and V65, reactive with an unknown epitope shared by all γ/δ TCR (Kühnlein, et al., 1994). As in other species, the two TCR isoforms are expressed in a mutually exclusive fashion on peripheral T-cells and thymocytes (Kühnlein et al., 1994). In order to obtain information on the lineage relationship between rat α/β and γ/δ T-cells, we first investigated the expression of TCRβ mRNA in γ/δ T-cell hybridomas.

RT-PCR analysis was performed on mRNA obtained from 9 γ/δ T-cell hybridomas using primers corresponding to rat Vβ and Cβ sequences. Whereas Cβ transcripts were readily detected in all cell lines analyzed, Vβ-Cβ amplificates were only found in 2 of the 9 γ/δ T-cell hybridomas investigated, although control experiments confirmed the capacity of the primers covering all 21 known rat Vβ segments containing a start codon and an open reading frame to amplify cDNA derived from α/β T-cells (data not shown). Sequence analysis of the two Vβ-Cβ amplificates obtained showed that they contained in-frame rearrangements. In one γ/δ hybridoma, Vβ13 had been joined to Jβ1.6 via an N nucleotide-flanked Dβ1 segment, whereas the other expressed an in-frame Vβ9-Dβ1-N-Jβ1.2 rearrangement (sequences not shown).

### Intracellular expression of TCRβ protein in α/β and γ/δ T-cells

In order to establish a tool for the enumeration of γ/δ T cells expressing functional TCRβ mRNA, we tested whether the TCR Cβ-specific mAb R73, known to also bind isolated β chains, would detect intracellular TCRβ protein. As shown in Fig. 1, fixation and permeabilization of α/β T-cell blasts readily allowed visualization of intracellular TCRβ chains by two-color flow cytometry. Interestingly, a significant fraction of γ/δ T-cell blasts (about 14 % of γ/δ cell surface positive cells) also reacted with the R73 mAb after the cells were both fixed and permeabilized. Since translation of TCR Cβ sequences can only proceed from mRNA transcribed from productively rearranged TCRβ genes, this result indicates that 1/7 of the γ/δ T-cell blasts contained TCRβ protein encoded by functionally rearranged Tcrb loci.

In order to investigate whether intracellular expression of TCRβ protein occurs *in vivo*, γ/δ T-cells from LEW lymph node, spleen and thymus were identified by cell surface staining with mAb V65, fixed and permeabilized, and counterstained with mAb R73. As shown in Fig. 2, roughly 15 % of γ/δ cells from each of the organs investigated expressed TCRβ chains intracellularly, in good agreement with the results obtained with activated γ/δ T-cell blasts. In these experiments, utilizing unseparated T-cells, a low frequency of apparently β-γ/δ "double positive" cells was already observed after fixing and prior to permeabilization (usually about 20% of the values obtained after permeabilization). This background is most likely due to the formation of α/β - γ/δ T-cell doublets during the fixation procedure, because it was not observed in purified γ/δ cells (Fig. 1).

### Kinetics of RAG-1 and γ mRNA expression in rat α/β T-cell maturation

The observed expression of i.c. TCRβ protein in γ/δ T-cells suggests that TCRβ expression by itself does not immediately and irreversibly commit maturing thymocytes to the α/β subset. In order to obtain evidence for γ/δ-specific gene expression in maturing thymocytes already expressing TCRβ, we turned to an *in vitro* system of thymocyte development.

As in other species, rat intrathymic α/β T-cell development procedes from a TN, that is TCR and coreceptor-negative, via an iSP, in this case $CD4^-8^+$ (Paterson et al., 1987), to the DP $CD4^+8^+$ stage, from which mature CD4 and CD8 T-cells are selected. Although the distinct maturational stages within the TN subset defined in mice (Godfrey et al., 1993) cannot be phenotypically identified in rats, the selective expression of the CD53 cell surface antigen on TN and on the mature thymocyte subsets allows purification of rat iCD8SP thymocytes by depletion of all other subsets with CD53- and CD4-specific mAb (Paterson et al., 1987). These transitional iCD8SP thymocytes are cycling cells (Paterson and Williams, 1987) which express a low level of cell-surface TCRβ chains (Hünig, 1988), presumably in conjunction with pre-Tα, and *in vitro* spontaneously and quantitatively convert to "virgin" DP thymocytes with α/β TCR cell-surface expression (Hünig and Mitnacht, 1991).

In order to investigate whether transitional rat iCD8SP thymocytes and their *in vitro*-generated $CD4^+8^+$ progeny expressed TCRγ transcripts, their mRNA was analyzed by RNAse protection for Cγ-specific sequences. In addition, a rat RAG-1 cDNA fragment was cloned and used as an antisense

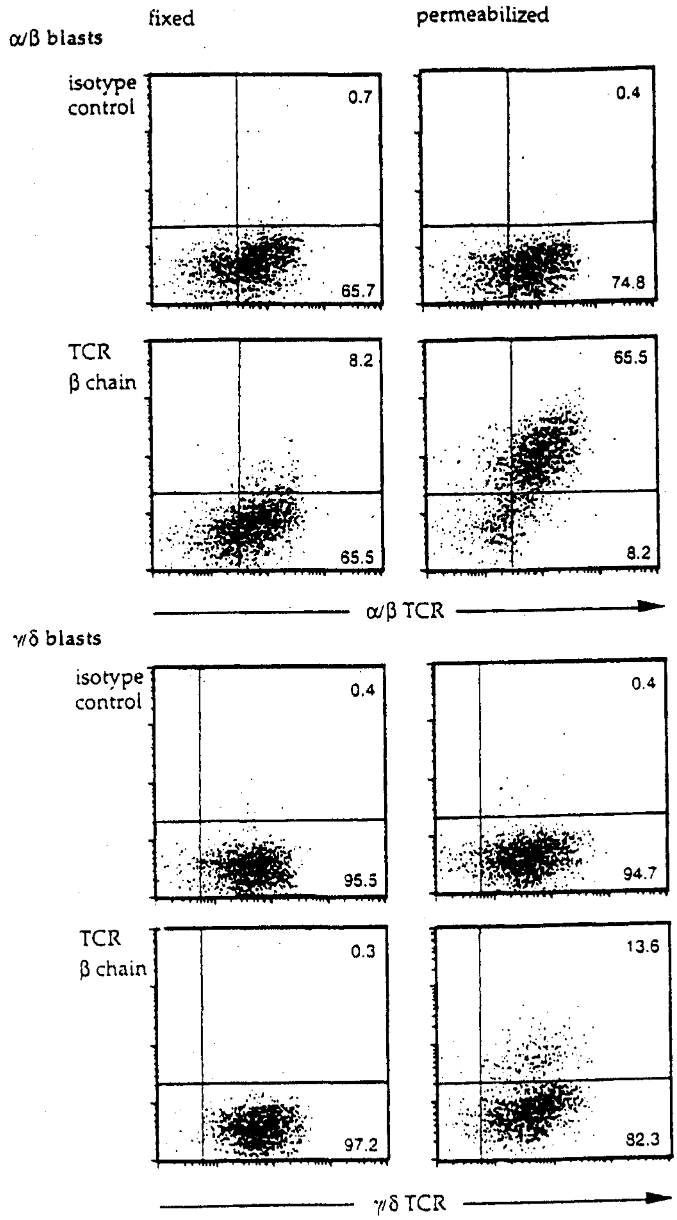

FIGURE 1 Detection of i.c. TCRβ protein in α/β and γ/δ T-cell blasts. T-cell blasts obtained by panning and expansion in cytokine-supplemented medium were surface stained with TCRβ-specific mAb R73 and TCRγ/δ-specific mAb V65, respectively, fixed (left column), or fixed and permeabilized (right column), before counterstaining for intracellular TCRβ expression with mAb R73 or an isotype control mAb probe to obtain information on the activation of the rearrangement machinery during this transition. As shown in Fig. 3B, the kinetics of RAG-1 expression observed closely follow results obtained in mice (reviewed by Fehling and von Boehmer, 1997): The transitory iSP subpopulation isolated *ex vivo* contained very little RAG-1 mRNA but strongly upregulated expression of this gene on entry into the DP

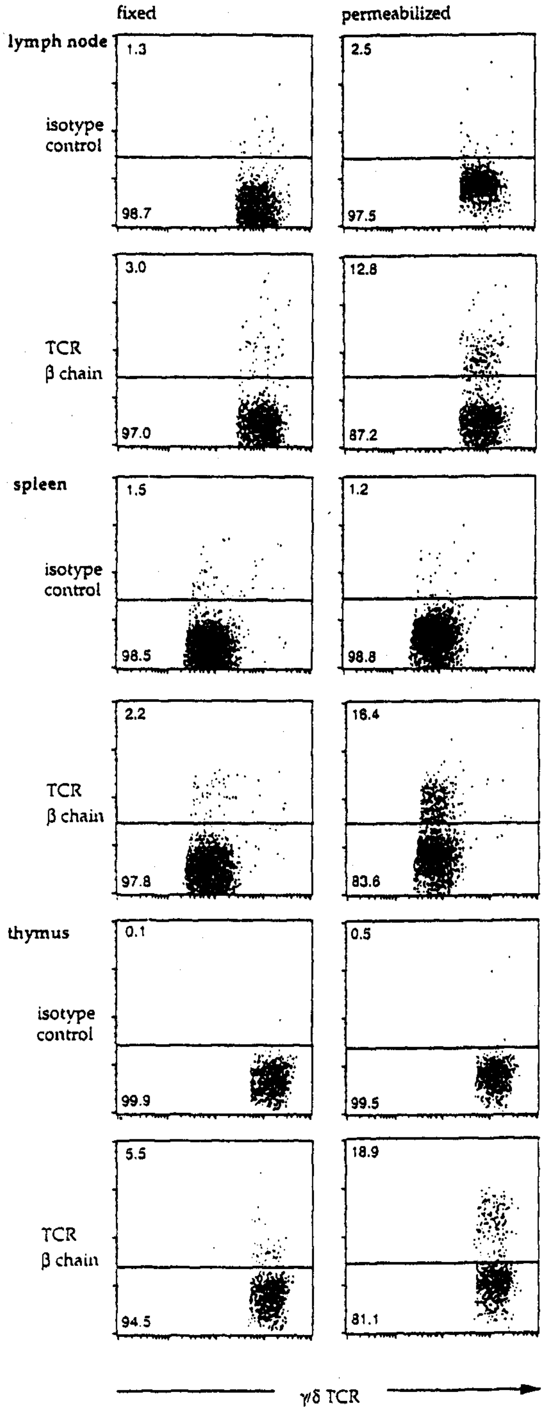

FIGURE 2 Detection of i.c. TCRβ protein in γ/δ T-cells *ex vivo*. Thymocytes or nylon wool passed lymph node or spleen cells were stained for surface expression of TCRγ/δ and intracellular expression of TCRβ, as described in the caption to Fig. 1. Events were collected after gating for TCRγ/δ-positive cells compartment. These results fit the model established for mouse α/β T-cell development, in which TCRβ rearrangement and expression precede entry of transitory late TN and iSP thymocytes expressing a pTCR consisting of a functional β chain and an invariant pTα chain but no rearrangement activity, into the $CD4^{+}8^{+}$ compartment with concomitant reactivation of the rearrangement machinery resulting in TCRα rearrangement and expression (Wilson et al., 1994, reviewed by Fehling and von Boehmer, 1997).

Interestingly, TCR Cγ transcripts were detectable at a low level in iCD8SP cells but not in their DP progeny obtained by overnight incubation (Fig. 3A). Since, at the same time, RAG-1 transcripts increased and the newly differentiated DP cells initiated cell-surface αβTCR expression (not shown; see Hünig and Mitnacht, 1991), the selective loss of Cγ mRNA suggests that lineage commitment is complete when the DP compartment is reached, in agreement with the silencing of TCRγ transcription at that stage in mice (Wilson et al., 1994, 1996). In addition, the presence of Cγ transcripts in iCD8SP cells indicates that either the potential for γ/δ differentiation is maintained in this subset *in vivo* but does not proceed in suspension culture or that lineage decision had occurred at the immediately preceding stage of differentiation, that is late TN cells, resulting in residual γ mRNA in the population analyzed. In any case, coexpression of TCR β and γ in iCD8SP cells supports the idea that lineage divergence can occur up to a late stage of pre-DP thymocyte differentation, in agreement with the intracellular expression of TCRβ protein in a subset of peripheral γ/δ T-cells.

### Intracellular Expression of TCRβ Protein in γ/δ T-cells from Euthymic and Athymic Rats

The well-established sequence of early differentiation events outlined earlier for the thymus has not been described for extrathymic T-cell development.

In order to assess whether intracellular expression of TCRβ chains in γ/δ T-cells is the result of common differentiation steps restricted to the thymus, nylon wool passed spleen and lymph node cells from 3 age-matched LEW, and congenic LEW *rnu/rnu* rats

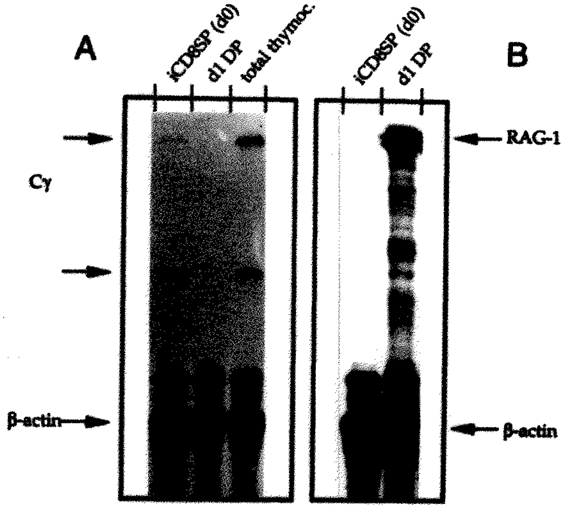

FIGURE 3 Kinetics of RAG-1 and γ mRNA expression during *in vitro* differentiation of transitory iCD8SP rat thymocytes. Highly purified iCD8SP thymocytes were obtained by depletion of all cells expressing CD4 and/or CD53, and used for isolation of cytoplasmic RNA either immediately or after incubation in culture medium for 1 day (d1DP). Spontaneous differentiation to DP cells is complete on d1. RNAse protection analysis using a Cγ (panel A) or a RAG-1 (panel B) antisense probe was performed as described in Materials and Methods were analyzed for the coexpression of cell-surface γ/δ TCR with intracellular β chains. As shown in Fig. 4 and table I, intracellular TCRβ protein was also present in γ/δ T-cells from athymic rats, although at only about half the frequency of that found in the euthymic animals analyzed in parallel. Therefore, rearrangement and expression of functional TCRβ chains in the γ/δ lineage does not depend on intrathymic T-cell development. However, control of lineage separation apparently differs in intra- versus extrathymic T-cell differentiation.

### DISCUSSION

The intracellular detection of TCRβ protein by flow cytometry has provided a powerful tool for the direct measurement of the frequency of γ/δ T-cells expressing a functionally rearranged β chain. Although the 15% value obtained may be a minimum estimate, the

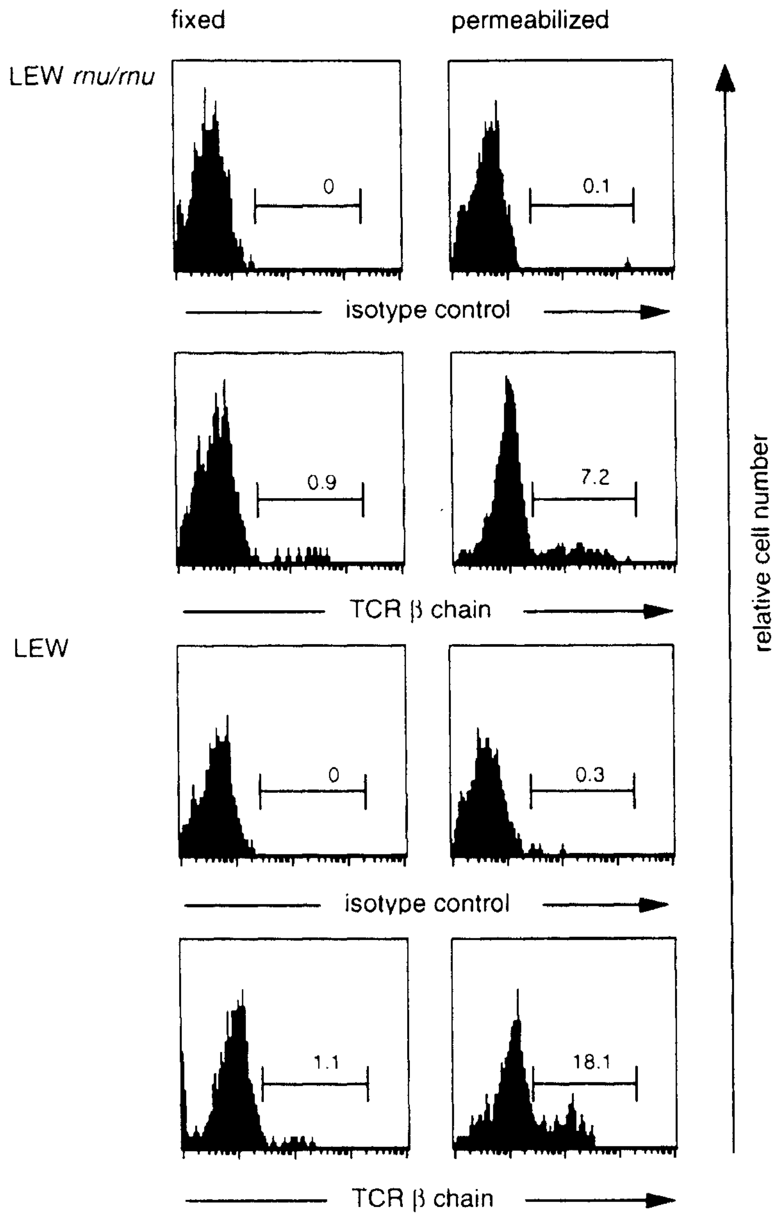

FIGURE 4 Expression of i.c. TCRβ protein in T-cells from athymic rats. Nylon wool-passed spleen and lymph node cells from age-matched individual euthymic LEW and athymic LEW *rnu/rnu* rats were analyzed for i.c. expression of TCRβ protein as described in the caption to Fig. 1. Histograms show cells gated for surface γ/δ TCR expression clear separation of positive and negative cells in this assay suggests that it detects all cells containing intracellular TCRβ protein. In support of this conclusion, two studies that appeared while this manuscript was under review report very similar frequencies for intracellular TCRβ expression in mouse γ/δ T-cells, that is 15 (Wilson et al., 1998) and 11 % (Aifantis et al., 1998), respectively.

TABLE I Expression of Intracellular TCRβ =Chains in γ/δ T-Cells from Euthymic and Athymic Rats

| *Strain* | *rat Number* | *% i.c.TCRβ⁺ of Surface γ/δ⁺ Cells*[a] | *Mean ± SD* |
|---|---|---|---|
| LEW | 1 | 16.9 | |
| | 2 | 18.1 | 16.5 ± 1.8 |
| | 3 | 14.5 | |
| LEW *rnu*/*rnu* | 1 | 10.7 | |
| | 2 | 10.0 | 9.3 ± 1.8 |
| | 3 | 7.2 | |

a. Note: Cells stained by mAb R73 minus isotype control (less than 0.2 %).
Note: Pooled nylon wool-passed spleen and lymph node cells were surface labeled with mAb V65, fixed, permeabilized, and counterstained with mAb R73

Unless one assumes that some γ/δ T-cells terminate β expression after maturation and others do not, the presence of TCRβ protein in 15% of γ/δ T-cells indicates that although β expression is permissive for the differentiation of γ/δ T-cells, $\beta^+$ precursors make only a minor contribution to the total γ/δ precursor pool. This agrees with findings in mice that γ/δ development appears unaffected in mice lacking either TCRβ or pre-Tα (Mombaerts et al., 1992; Fehling et al., 1995).

Have those γ/δ T-cells that express i.c. TCRβ been expanded by the proliferative burst associated with β selection? Recent data obtained in mice come to opposite conclusions: In one analysis, the frequency of cycling cells in i.c. $TCR\beta^+$ thymic γ/δ T-cells was twice as high as in those lacking TCRβ protein, supporting TCRβ-driven expansion (Wilson and MacDonald, 1998). In the other study, however, only a very small increase of cycling cells was seen in the i.c. $TCR\beta^+$ subset (Aifantis et al., 1998). This study also reported that abrogation of β selection (but not of β expression) by inactivation of the pTα gene increases the frequency of γ/δ T-cells expressing intracellular β protein, suggesting that the pTCR signal depletes TCRβ-expressing cells from the available γ/δ T-cell precursor pool by committing them to the α/β lineage.

Our present findings that TCRγ message is detectable in the latest stage of β selected cells, the cycling iCD8SP intermediates that quantitatively differentiate to DP cells after 16 h of suspension culture (Paterson and Williams, 1987; Hünig, 1988), but not in these "virgin" DP cells themselves, suggest that the potential to digress to the γ/δ T-cell lineage is already lost at the iCD8SP stage and that the expression of TCRγ message reflects ongoing lineage separation at the preceding, late TN stage. It cannot be excluded, however, that the suspension culture system employed lacks signals that would allow γ/δ T-cell differentiation at that stage *in vivo*.

We believe these comparisons between mouse and rat thymocyte development to be valid because the regulation of TCR and RAG expression during the transition from the TN (or iSP) to the DP compartment is identical in both species. Thus, RAG-1 transcription is shut down in β-selected cycling cells and reactivated on entry into the DP subset, preceding TCRα/β expression. Furthermore, in experiments presently not shown, we found that *in vitro* stimulation of these synchronously differentiating DP thymocytes with TCR-specific mAb leads to a very rapid disappearance of RAG-1 transcripts without interfering with TCRα and β mRNA levels (H.P. and T.H., unpublished). Thus, *in vitro* differentiation of rat iCD8SP cells and their response to TCR engagement very closely follow maturation events established in the mouse model (reviewed by Fehling and von Boehmer, 1997). The twofold reduction of i.c. TCRβ expression in γ/δ T-cells from athymic rats indicates that intra- and extrathymic control of α/β versus γ/δ development is not identical. At present, it is difficult to conclude whether this reflects a different impact of β selection in these two settings. In athymic mice, pre-Tα expression has been detected (Bruno et al.,

1995), but a cycling β-selected intermediate remains to be defined. Since in euthymic mice, the absence of pTα and hence of β selection leads to an enrichment of γ/δ cells with i.c. TCRβ expression, presumably because pTα expression instructs α/β commitment (Aifantis et al., 1998), the reduction of i.c. TCR β-expression in γ/δ cells from athymic as compared to euthymic rats may indicate that in extrathymic T-cell differentiation, β expression more rigorously commits precursors to the α/β lineage than in intrathymic T-cell development. Alternatively, the finding that intrathymic γ/δ cells with i.c. TCRβ expression show enhanced cycling as compared to their i.c. $TCR\beta^-$ counterparts (Wilson and MacDonald, 1998) raises the possibility that a lack of pTCR-driven numeric expansion outside the thymus reduces the contribution of i.c. $TCR\beta^+$ precursors to the γ/δ lineage. Finally, T-cell precursors may rearrange Tcrb at a lower frequency outside than inside the thymus. In order to distinguish between these possiblities, it will be of interest to see the impact of pTα deficiency on extrathymic T-cell development, including i.c. TCR β-expression in the γ/δ lineage.

The expression of TCRβ protein in 1/7 rat γ/δ T-cells raises the possibility that "mixed lineage" TCR heterodimers are formed. Interestingly, expression of a functional β/γ TCR recognized by the TCRβ-specific mAb R73 has been observed in a chemically induced rat thymic lymphoma (Kinebuchi et al., 1997). Among normal γ/δ T-cells, cell surface expression of this mixed TCR is, however, very rare or absent (Kühnlein et al., 1994). This suggests that once a functional δ chain is available, pairing with TCRβ is avoided by competition or an unknown interfering mechanism, and that potential immature precursors with β/γ TCR are rare, short-lived, and fail to be positively selected.

In summary, the present results demonstrate flexibility of α/β-γ/δ lineage divergence in early steps of rat thymocyte differentiation that is terminated by commitment after Tcrb rearrangement and before entry of α/β lineage cells into the DP compartment. Furthermore, they provide a first estimate of TCRβ protein expression in γ/δ cells based on a single-cell assay, which is likely to reflect the frequency of cells carrying a productive β rearrangement. The value obtained (about 15%) indicates that successful β rearrangement is permissive but not mandatory for γ/δ T-cell development. It remains to be seen whether in addition to this small quantitative effect, β expression has an impact on the quality of the γ/δ repertoire through an expansion of those precursors that represent the latest possible point of divergence from the α/β lineage, facilitating secondary TCR rearrangements through an increased number of cell divisions.

### MATERIALS AND METHODS

### Rats, Cell Lines and Antibodies

Young adult Lewis (LEW) rats of both sexes were obtained from the institute's colony. Athymic LEW/Mol *rnu/rnu* rats and euthymic controls were obtained from Mollegaard Breeding and Research Center, Denmark. α/β and γ/δ T-cell hybridomas were prepared from ConA-activated splenic T-cell blasts fused with a TCRα/β-deficient variant of the mouse BW5147 thymoma (White et al., 1989). mAbs W3/25 and OX-35 (both anti-CD4), 341 (anti-CD8β), OX-44 (anti-CD53), and R73 (anti-TCRα/β), were obtained from Pharmingen (San Diego, CA), and from Serotec (Oxford, U.K.) as purified antibody or antibody conjugates. PE-conjugated $F(ab)_2$donkey anti-mouse Ig was obtained from Dianova GmbH (Hamburg, Germany).

### Preparation and Culture of Cells

T cells were enriched from pooled superficial and mesenteric lymph nodes or from spleen by passage over nylon wool. Purified α/β and γ/δ T-cell blasts were prepared as described by panning of nylon wool passed cell suspensions in mAb R73- or V65-coated tissue culture flasks and expanding the adherent cells in medium containing a cytokine cocktail (Kühnlein et al., 1994). After 3 days, cells were harvested by vigorous pipetting and cultured for another 4 hours to allow TCR cell-surface reexpression.

iCD8SP cells were prepared from thymocyte suspensions by depleting CD4-and/or CD53-expressing cells by rosetting and immunomagnetic separation as described (Hünig and Mitnacht, 1991). The resulting population (over 98% pure, devoid of detectable γ/δ or α/β $TCR^+$ cells) was cultured at $2 \times 10^6$ cells/ml in complete medium (Hünig et al., 1989) without stimulation.

### Immunofluorescence and Flow Cytometry

Three-color analysis of cell-surface expression of TCR, CD4, and CD8 in developing thymocytes was performed as previously described (Itano et al., 1996), using a FACScan flow cytometer, LYSYS II software for acquisition, and Cellquest software for analysis (all from Becton Dickinson, Mountainview, CA). Dot plots are shown as $\log_{10}$ fluorescence intensities on a four-decade scale. For intracellular staining (Kraus et al., 1992), $5 \times 10^5 - 2 \times 10^6$cells were first surface labeled either with directly conjugated mAb or indirectly via PE-conjugated $F(ab)_2$donkey anti-mouse Ig followed by blocking with normal mIg, washed twice and then fixed in ice-cold formalin (0.5% in PBS without $Ca^{2+}$ and $Mg^{2+}$) for 30 min on ice. After two washing steps with FACS buffer, the fixed cells were permeabilized in 4 mg/ml n-octyl-β-D-glucopyranoside (Sigma) in 0.13 M $Na_2HPO_4$, 0.02 M $NaH_2PO_4$, 0.14 M NaCl, incubated at RT for 7 min and washed twice. To block unspecific binding, the cells were resuspended in 0.025M Tris supplemented with 10% FCS and left on ice for 20 min before directly fluorochrome-conjugated mAb directed against the intracellular antigen was added. Flow cytometry and analysis were performed as described earlier.

### RNAse Protection Analysis

Isolation of cytoplasmic RNA and RNase protection assays were performed as previously described (Park et al., 1993). A cDNA clone encoding TCRγ from the AO rat strain was kindly provided by A. Neil Barclay (Oxford, UK) (Morris et al., 1988). C and 3'UT sequences were subcloned into pGEM-3Z (Promega Corp., Madison, WI). The double band observed with the antisense TCRγ cDNA fragment is highly reproducible and presumably reflects a polymorphism between AO and LEW. A 513-bp fragment of the rat RAG1 cDNA was cloned by PCR amplification of rat thymocyte cDNA using a 21-mer upstream primer corresponding to the human sequence terminating at the start codon, and a 20-mer downstream primer derived from the mouse 475–495 sequence. The amplified PCR product was cloned into pBluescript II KS+ vector (Stratagene, La Jolla, CA), sequenced, and found to display 92% and 87% identity to the human and mouse sequences, respectively (EMBL gene bank accession no. AJ006070). The transcription vector pSPbact72 constructed by M. Jantzen containing a fragment of the rat β-actin cDNA was kindly provided by F. Siebelt (Würzburg). T7 and SP6-driven transcription was used to generate $^{32}P$-labeled antisense probes.

### PCR Analysis

RNA was isolated by ethanol precipitation from cytoplasmic Nonidet P-40 extracts following the method described by Gough (1988). 1 μg RNA was converted into cDNA using 0.1 μg oligo dT primer (Gibco/BRL) and 100 U MMLV reverse transcriptase (Gibco/BRL) according to manufacturer's recommendations. For PCR amplification, the following primers were used: 1) sense Vβ primers were 18 to 21 mers initiating at the start codon of the 21 known rat Vβ genes containing an ATG start codon and an open reading frame (Smith et al., 1991) (2) antisense Cβ (5'TTC AGG AAC TCT TTC TTT TGA C3') used with the Vβ primers to generate Vβ-Cβ amplificates; or with (3) sense Cβ (5'ATA TAC ATA TGG AGG ATC TGA AAA CGG TGA CT3') for Cβ amplificates.

The PCR reaction mixture contained 1 μl cDNA, 10 μM of each primer, 100 μM of each dNTP in Taq Polymerase buffer (50 mM KCl, 10 mM Tris-HCl, pH 9, 1.5 mM $MgCl_2$, 0.1% Triton X-100). The samples were overlaid with mineral oil (Sigma), heated to 94°C for 5 min before adding 1 U Taq DNA polymerase (MBI Fermentas) and subjected to 30 amplification cycles of 1 min at 94°C for denaturing, 1 min at 56°C for annealing, and 1 min, 10 sec at 72°C for elongation. The last cycle was followed by a 10-min elongation at 72°C.

### *Acknowledgements*

This work was supported by the DFG through SFBs 165 and 465, and by Fonds der Chemischen Industrie e.V. We thank Neil Barcley for the TCR γ containing plasmid, Gerhard Giegerich for providing Cβ-specific primers, Kathrin Hoffmann for photography, and Anneliese Schimpl and Thomas Herrmann for helpful discussions.

### *References*

Aifantis I., Azogui O., Feinberg J., Saint-Ruf C., Buer J., and von Boehmer H. (1998). On the role of the pre-T cell receptor in alphabeta versus gammadelta T lineage commitment. Immunity **9**: 649–655.

Bruno, L., Rocha, B., Rolink, A., von Boehmer, H. and Rodewald, H. R. (1995). Intra- and extra-thymic expression of the pre-T cell receptor alpha gene. Eur. J. Immunol. **25**: 1877–1882.

Burtrum D. B., Kim S., Dudley E. C., Hayday A. C., and Petrie H. C. (1996). TCR gene recombination and αβ-γδ lineage divergence: Productive TCR-β rearrangement is neither exclusive nor preclusive of gd cell development. J. Immunol. **157**: 4293–4296.

Chien Y. H., Iwashima M., Kaplan K. B., Elliott J. F. and Davis M. M. (1987a). A new T-cell receptor gene located within the alpha locus and expressed early in T-cell differentiation. Nature **327**: 677–682.

Chien Y. H., Iwashima M., Wettstein D. A., Kaplan K. B., Elliott J. F., Born W., and Davis M. M. (1987b). T-cell receptor delta gene rearrangements in early thymocytes. Nature **330**: 722–727.

Dudley E. C., Girardi M., Owen M. J. and Hayday A. C. (1995). Alpha, beta, and gamma delta T cells can share a late common precursor. Curr Biol. 5: 659–669.

Dudley E. C., Petrie H. T., Shah L. M., Owen M. J., and Hayday A. C. (1994). T cell receptor beta chain gene rearrangement and selection during thymocyte development in adult mice. Immunity **1**: 83–93.

Fehling H. J., Krotkova A., Saint-Ruf D., and von Boehmer H. (1995). Crucial role of the pre-T-cell receptor α gene in development of αβ but not γ/δ T cells. Nature **375**: 795–798.

Fehling H. J., and von Boehmer H. (1997). Early alpha beta T cell development in the thymus of normal and genetically altered mice. Curr. Opin. Immunol. **9**: 263–275.

Fowlkes B. J., Edison L., Mathieson B. J., and Chused T. M. (1985). Early T lymphocytes. Differentiation in vivo of adult intrathymic precursor cells. J. Exp. Med. **162**: 802–822.

Garman R. D., Doherty P., and Raulet D. H. (1986). Diversity, rearrangement and expression of murine T-cell gamma genes. Cell **45**: 733–742.

Godfrey D. I., Kennedy J., Suda T., and Zlotnik A. (1993). A developmental pathway involving four phenotypically and functionally distinct subsets of CD3-CD4-CD8- triple-negative adult mouse thymocytes defined by CD44 and CD25 expression. J. Immunol. **150**: 4244–4252.

Gough N. M. (1988). Rapid and quantitative preparation of cytoplasmic RNA from small numbers of cells. Anal. Biochem. **173**: 93–95.

Havran W. L., and Allison J. P. (1988). Developmentally ordered appearance of thymocytes expressing different T-cell antigen receptors. Nature **335**: 443–445.

Hünig T. (1983). T-cell function and specificity in athymic mice. Immunol. Today **4**: 84–87.

Hünig T. (1988). Crosslinking of the T-cell antigen receptor interferes with the generation of CD4+8+thymocytes from their immediate CD4–8+ precursors. Eur. J. Immunol. **18**: 2089–2092.

Hünig T., and Mitnacht R. (1991). T cell receptor-mediated selection of functional rat CD8 T-cells from defined immature thymocyte precursors in short-term suspension culture. J. Exp. Med. **173**: 561–568.

Hünig T., Tiefenthaler G., Lawetsky A., Kubo R., and Schlipköter E. (1989). T-cell subpopulations expressing distinct forms of the TCR in normal, athymic, and neonatally TCR alpha beta-suppressed rats. Cold Spring Harb. Symp. Quant. Biol. **54**: 61–68.

Hünig T., Wallny H.-J., Hartley J. K., Lawetzky A., and Tiefenthaler G. (1989). A monoclonal antibody to a constant determinant of the rat T cell antigen receptor that induces T cell activation. J. Exp. Med. **169**: 73–86.

Itano A., Salmon P., Kioussis D., Tolaini M., Corbella P., and Robey E. (1996). The cytoplasmic domain of CD4 promotes the development of CD4 lineage T cells. J. Exp. Med. **183**: 731–741.

Itohara S., Nakanishi N., Kanagawa O., Kubo R., and Tonegawa S. (1989). Monoclonal antibodies specific to native murine T-cell receptor γδ: Analysis of γδ T cells during thymic ontogeny and in peripheral lymphoid organs. Proc. Natl. Acad. Sci. USA **86**: 5094–5098.

Kang J., Baker J., and Raulet D. H. (1995). Evidence that productive rearrangements of TCR gamma genes influence the commitment of progenitor cells to differentiate into alpha beta or gamma delta T cells. Eur. J. Immunol. **25**: 2706–2709.

Kang J., and Raulet D. H. (1997). Events that regulate differentiation of alpha beta TCR+ and gamma delta TCR+ T cells from a common precursor. Semin. Immunol. **9**: 171–179.

Kinebuchi M., Matsuura A., Ogiu T., and Kikuchi K. (1997). Deviated overexpression TCR-β, TCR-γ, CD4 and CD8 on thymic lymphomas induced by 1-propyl-1-nitrosurea. J. Immunol. **159**: 748–756.

Kraus E., Schneider-Schaulies S., Miyasaka M., Tamatani T., and Sedgwick J. (1992). Augmentation of major hisocompatibility complex class I and ICAM-1 expression on glial cells following measles virus infection: Evidence for the role of type-1 interferon. Eur. J. Immunol. **22**: 175–182.

Kühnlein P., Mitnacht R., Torres-Nagel N. E., Herrmann T., Elbe A., and Hünig T. (1996). The canonical T-cell receptor of dendritic epidermal γ/δ T-cells is highly conserved between rats and mice. Eur. J. Immunol. **26**: 3092–3097.

Kühnlein P., Park H.-J., Herrmann T., Elbe A. and Hünig T. (1994). Identification and characterization of rat γ/δ T lymphocytes in peripheral lymphoid organs, small intestine, and skin with a monoclonal antibody to a constant determinant of the γ/δ T-cell receptor. J. Immunol. **153**: 979–986.

Kühnlein P., Vicente A., Varas A., Hünig T. and Zapata A. (1995). γ/δ T cells in fetal, neonatal, and adult rat lymphoid organs. Dev. Immunol. **4**: 181–188.

Lake J. P., Pierce C. W. and Kennedy J. D. (1991). T cell receptor expression by T cells that mature extrathymically in nude mice. Cell. Immunol. **135**: 259–265.

Lawetzky A., Tiefenthaler G., Kubo R., and Hünig T. (1990). Identification and characterization of rat T-cell subpopulations expressing α/β and γ/δ T-cell receptors. Eur. J. Immunol. **20**: 343–349.

Livac F., Petrie H. T., Crispe I. N., and Schatz D. G. (1995). In-frame Tcr d gene rearrangements play a critical role in the αβ/γδ lineage decision. Immunity **2**: 617–627.

Malissen M., Trucy J., Jouvin Marche E., Cazenave P. A., Scollay R., and Malissen B. (1992). Regulation of TCR alpha and beta gene allelic exclusion during T-cell development. Immunol. Today **13**: 315–322.

Mallick C. A., Dudley E. C., Viney J. L., Owen M. J., and Hayday A. C. (1993). Rearrangement and diversity of T cell receptor beta chain genes in thymocytes: A critical role for the beta chain in development. Cell **73**: 513–519.

Matis L. A., Cron R., and Bluestone J. A. (1987). Major histocompatibility complex-linked specificity of gamma delta receptor-bearing T lymphocytes. Nature **330**: 262–264.

Mombaerts P., Clarke A. R., Rudnicki M. A., Iocomini J., Itohara S., Lafaille J. J., Wang L., Ichikawa Y., Jaenisch R., Hooper M. L., and Tonegawa S. (1992). Mutations in the T-cell antigen receptor genes α and β block thymocyte development at different stages. Nature **360**: 225–331.

Morris M., Barclay A. N., and Williams A. F. (1988). Analysis of T cell receptor β chains in rat thymus, and rat Cα and Cβ sequences. Immunogenetics **27**: 174–179.

Nakajima P. B., Menetski J. P., Roth D. B., Gellert M., and Bosma M. J. (1995). V-D-J rearrangements at the T cell receptor delta locus in mouse thymocytes of the alpha beta lineage. Immunity **3**: 609–621.

Park J.-H., Mitnacht R., Torres-Nagel N., and Hünig T. (1993). T cell receptor ligation induces interleukin (IL) 2Rβ chain expression in rat CD4,8 double positive thymocytes, initiating an IL-2-dependent differentiation pathway of $CD8a^+/b^-$ T-cells. J. Exp. Med. **177**: 541–546.

Paterson D. J., Green J. R., Jefferies W. A., Puklavec M., and Williams A. F. (1987). The MRC OX-44 antigen marks a functionally relevant subset among rat thymocytes. J. Exp. Med. **165**: 1–13.

Paterson D. J., and Williams A. F. (1987). An intermediate cell in thymocyte differentiation that expresses CD8 but not CD4 antigen. J. Exp. Med. **166**: 1603–1608.

Petrie H. T., Scollay R., and Shortman K. (1992). Commitment to the T cell receptor-alpha beta or-gamma delta lineages can occur just prior to the onset of CD4 and CD8 expression among immature thymocytes. Eur. J. Immunol. **22**: 2185–2188.

Robey E., and Fowlkes B. J. (1998). The αβ vesus γδ T-cell lineage choice. Curr. Opin. Immunol. **10**: 181–187.

Saito H., Kranz D. M., Takagaki Y., Hayday A. C., Eisen H. N., and Tonegawa S. (1984). A third rearranged and expressed gene in a clone of cytotoxic T lymphocytes. Nature **312**: 36–40.

Scollay R., Wilson A., D'Amico A., Kelly K., Egerton M., Pearse M., Wu L., and Shortman K. (1988). Developmental status and reconstitution potential of subpopulations of murine thymocytes. Immunol. Rev. **104**: 81–120.

Shinkai Y., Rathbun G., Lam K. P., Oltz E. M., Stewart V., Mendelsohn M., Charron J., Datta M., Young F., Stall A. M., and Alt F.W. (1992). RAG-2-deficient mice lack mature lymphocytes owing to inability to initiate V(D)J rearrangement. Cell **68**: 855–867.

Smith L. R., Kono D. H., and Theofilopolous A. N. (1991). Complexity and sequence identification of 24 rat Vβ genes. J. Immunol. **147**: 375–379.

Vicari A. P., Mocci S., Openshaw P., O'Garra A., and Zlotnik A. (1996). Mouse gamma delta TCR+NK1.1+ thymocytes specifically produce interleukin-4, are major histocompatibility complex class I independent, and are developmentally related to alpha beta TCR+NK1.1+ thymocytes. Eur. J. Immunol. **26**: 1424–1429.

White J., Herman A., Pullen A. M., Kubo R., Kappler J. W., and Marrack P. (1989). The Vb-specific superantigen Staphylococcal enterotoxin B: Stimulation of mature T cells and clonal deletion in neonatal mice. Cell **56**: 27–35.

Wilson A., de Villartay J. P., and MacDonald H. R. (1996). T cell receptor delta gene rearrangement and T early alpha (TEA) expression in immature alpha beta lineage thymocytes: Implications for alpha beta/gamma delta lineage commitment. Immunity **4**: 37–45.

Wilson A., Held W., and MacDonald H. R. (1994). Two waves of recombinase gene expression in developing thymocytes. J. Exp. Med. **179**: 1355–1360.

Wilson A., and MacDonald H. R. (1998). A limited role for beta-selection during gamma delta T cell development. J. Immunol. **161**: 5851–5854.

